# Retraction: Long non-coding RNA ROR1-AS1 incudes tumorigenesis of colorectal cancer by affecting Wnt/β-catenin signaling pathway

**DOI:** 10.1042/BSR-20191453_RET

**Published:** 2020-07-17

**Keywords:** colorectal cancer, Long noncoding RNA, ROR1-AS1, Wnt/β-catenin signaling pathway

This Retraction follows an Expression of Concern relating to this article previously published by Portland Press.

This article is being retracted from Bioscience Reports at the request of the authors following receipt of a notification from a reader, alerting the Journal to multiple duplicated images in Figure 2, 3C, 3D, 3E and 4B. The Editorial Office has also been made aware that these images have been reused in multiple publications, which brings into question the validity of the data presented in this paper.

The authors wish to retract the article. The Editor-in-Chief and Editorial Board agree with the retraction.

